# Supplementary material for: The interactome of intact mitochondria by cross-linking mass spectrometry provides evidence for coexisting respiratory supercomplexes
Source: Mol Cell Proteomics. 2017 Dec 8;17(2):216–32. doi: 10.1074/mcp.RA117.000470 (PMC5795388; doi:10.1074/mcp.RA117.000470)
Supplement: Supplemental Data [file supp_RA117.000470_133922_0_supp_23978_fzffwf.zip › spectra_annotation/mito_DR_spectra_annotation/90-1-3-1-20-1.pdf]

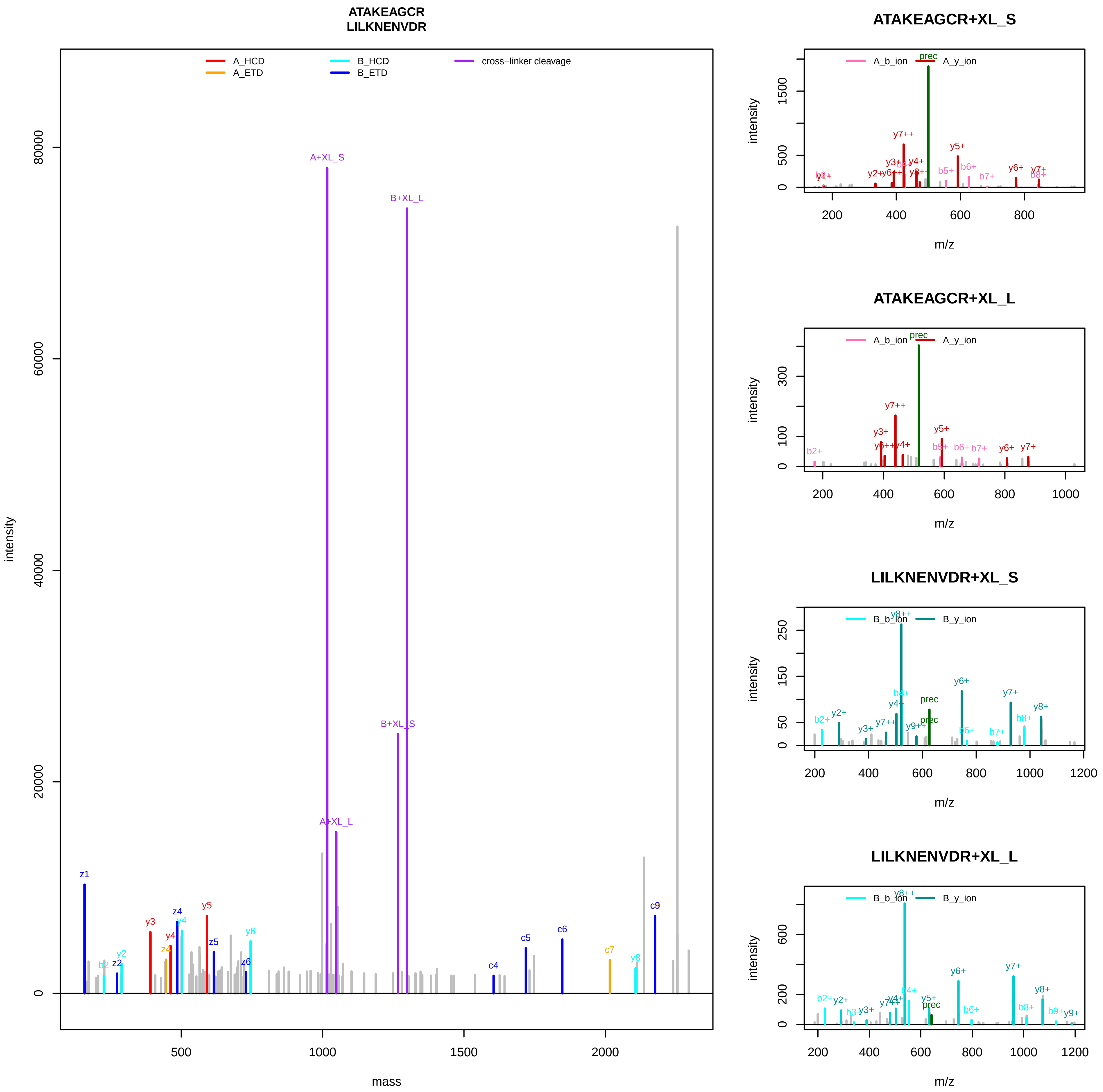
ATAKEAGCR
LILKNENVDR
A_HCD
A_ETD
B_HCD
B_ETD
cross−linker cleavage
A+XL_S
B+XL_L
B+XL_S
A+XL_L
z1
b2
z2
y2
y3
z4
y4
z4
y4
y5
z5
z6
y6
c4
c5
c6
c7
y8
c9
intensity
mass
0
20000
40000
60000
80000
500
1000
1500
2000
ATAKEAGCR+XL_S
ATAKEAGCR+XL_L
LILKNENVDR+XL_S
LILKNENVDR+XL_L
A_b_ion
A_y_ion
B_b_ion
B_y_ion
prec
y7++
y5+
y6+
y7+
b6+
b7+
b8+
y3+
y4+
b5+
b2+
y2+
y8+
b4+
y9+
b9+
m/z
0
500
1500
100
300
50
150
250
200
600
200
400
600
800
1000
1200
